# Knowledge, attitudes, and practices toward halitosis among dental patients at Zewditu memorial hospital, Addis Ababa, Ethiopia

**DOI:** 10.3389/froh.2025.1522682

**Published:** 2025-02-24

**Authors:** Rediet Degif, Yeshewas Abaynew

**Affiliations:** ^1^ Department of Dental Medicine, Atlas College of Health Sciences, Addis Ababa, Ethiopia; ^2^ School of Public Health, College of Medicine and Health Sciences, Wollo University, Dessie, Ethiopia

**Keywords:** knowledge, attitude, practice, halitosis, bad breath

## Abstract

**Background:**

Bad breath, or halitosis, is a common oral health problem that can significantly affect a person's social and psychological well-being. Understanding dental patients' knowledge, attitudes, and practices related to halitosis provides valuable insights into the current state of KAP related to halitosis in Addis Ababa and highlights the need for targeted educational interventions within dental health services.

**Objective:**

This study aimed to assess the knowledge, attitudes, and practices toward halitosis among dental patients at Zewditu Memorial Hospital in Addis Ababa, Ethiopia.

**Methods:**

An institution-based cross-sectional study design was employed. A systematic random sampling technique was used to select 214 study participants. Data were collected via a structured questionnaire that assessed the participants’ knowledge, attitudes, and practices related to halitosis. The data were checked, cleaned, and entered into SPSS version 25.0. Descriptive statistics were used to describe the data, and the findings were presented in texts, tables, and graphs.

**Results:**

In terms of knowledge, 71% of the participants had poor knowledge about halitosis. Approximately 68.7% of the participants had an unfavorable attitude toward halitosis. Furthermore, while 40.7% of the participants reported regular oral care and 42.5% used mouthwash, only 26.6% of the participants reported good practices regarding halitosis.

**Conclusion:**

Overall, knowledge, attitudes, and practices regarding halitosis among dental patients at Zewditu Memorial Hospital were not satisfactory. These results highlight critical gaps in public awareness and effective management strategies for halitosis. This study highlights the need for integrated public health initiatives and clinical practices that focus on improving awareness and management of halitosis to ultimately promote better oral health in the community.

## Introduction

Halitosis is a noticeably unpleasant odor from the mouth that is unpleasant to others. It is the third most common reason for seeking dental care after tooth decay and periodontal disease (1). Oral halitosis can be attributed to a high local concentration of intraoral microbial populations, particularly biofilms on the tongue, associated with teeth and periodontal tissues.^1^ The classification of halitosis includes categories of genuine halitosis, pseudohalitosis, and halitophobia (2).

Halitosis occurs worldwide, with a prevalence of 22%–50% (3). Several studies have demonstrated that the oral cavity is the main cause of bad breath in 85%–90% of patients with halitosis (4). The main cause of intraoral halitosis is anaerobic microorganisms in the tongue coating (5, 6). It is not a serious life-threatening problem but has great value in social interaction (7).

Bad breath is associated with psychiatric symptoms such as phobias, depression, significant anxiety, and behavioral changes and can negatively affect self-esteem and self-confidence and impair social participation (8). The psychological effect has forced individuals to excessively use chewing gums, mouthwashes, or medications to mask odor and solve this distressing problem (9).

A study in Karachi, Pakistan, revealed that 54.7% of participants reported having bad breath (10). In Saudi Arabia (11), the population is aware that periodontal disease, decayed teeth, and foul-smelling food are the main causes of bad breath. However, other important causes of bad breath, such as tongue coating; dry mouth, ear, nose, and throat infections; and stomach problems, are not understood. In the Indian study, the majority of the population knew only tongue coating (35.5%) and food (40.5%) as possible causes of bad breath (12).

In a cross-sectional study of the general population, almost 90% of the participants in a Dutch survey indicated being faced with a person with halitosis regularly (13). In a study conducted in Dharan, Eastern Nepal, 13.6% of participants reported halitosis, 55.2% had adequate knowledge about halitosis, 50.1% had a positive attitude toward halitosis, and 54.3% of respondents had unsatisfactory practices related to halitosis (14).

In an Indian study of individuals' attitudes toward halitosis, approximately 52.5% of the respondents self-perceived halitosis to be self-reported, 76.2% had never visited a dentist, and 61.25% were not willing to see dentists. This depicts the negative attitude of the population toward halitosis (12).

In a study involving dental patients in Pakistan, 55% of individuals cleaned their teeth with toothpaste and miswak (wooden stick), 37.1% did not use toothpaste/miswak, and 7.9% used it occasionally to brush their teeth; thus, their practices were not satisfactory (15). In Benin, clinical students' practices toward halitosis were not encouraging (16).

Studies on halitosis are limited in Ethiopia, and a previous study in Northwest Ethiopia (1) reported that 44.2% of patients attending dental clinics presented signs of halitosis. Halitosis is a major but underestimated taboo, and assessment methods are not standardized; moreover, no universally accepted standard criteria define a halitosis disease (5, 17).

There is limited data on knowledge, attitudes, and practices (KAPs) related to halitosis in Ethiopia. Previous studies have examined various aspects of oral health, but few specific studies have focused on KAP related to halitosis in the Ethiopian context. This gap highlights the need for a baseline assessment to inform future interventions and educational programs. By establishing a baseline understanding of KAP toward halitosis, the findings can guide public health initiatives, inform healthcare professionals, and contribute to the development of targeted educational materials aimed at improving awareness and treatment of halitosis. This study investigated the knowledge, attitudes, and practices related to halitosis among dental patients at Zewditu Memorial Hospital.

## Materials and methods

### Study area and periods

The study was conducted at Zewditu Memorial Hospital (ZMH) in Addis Ababa. This study was conducted from February 11, 2024, to March 13, 2024.

### Study design

An institution-based cross-sectional study was used.

### Population

The source population was dental OPD patients at Zewditu Memorial Hospital, while the study population included dental patients who visited the dental OPD at Zewditu Memorial Hospital during the data collection period. Dental patients aged 18 years and older were included, whereas severely sick patients who did not cooperate during data collection were excluded from the study.

### Sample size determination and sampling method

The sample size of the study was calculated using a single population proportion formula, considering the following assumption: *p*, 50% (no previous studies on KAP); *d* = 5%; 95% confidence level, *Z* α/2 = 1.96. The previous month's report revealed that 396 patients who attended the dental outpatient department at Zewditu Memorial Hospital composed the study population, which was less than 10,000 population. A population correction formula was applied to obtain the sample size. Considering a 10% nonresponse rate, the final sample size was 214. Zewditu Memorial Hospital was purposively selected to obtain adequate dental patients for this study. The 214 study participants visiting the dental OPD at Zewditu Memorial Hospital were subsequently selected using systematic random sampling. Systematic sampling was used primarily to enhance logistical efficiency and ensure a more manageable sampling process. This method allows for a straightforward and organized selection of participants, which can be particularly beneficial in clinical settings with a high volume of patients. Study participants were included with an interval of 2 patients. The starting point for the sampling was randomly selected, which helps maintain the randomness of the sample.

### Data collection methods and tools

Data were collected via face-to-face interviews using a structured questionnaire covering socio-demographic characteristics, knowledge about halitosis, attitudes toward halitosis, and practices related to halitosis. The questions were developed on a literature review. For the attitude questions, a Likert scale with values ranging from 1 (strongly disagree) to 5 (strongly agree) was used. A pilot test was conducted with a small group of participants to assess the clarity and relevance of the questions, followed by adjustments based on their feedback. The questions were reviewed by experts for content validity. The reliability of the constructs was assessed using Cronbach's alpha and resulted in internal consistency for the knowledge items (0.82), attitudes (0.76), and practices (0.79). The questionnaire was developed in English and translated into Amharic.

### Data entry and analysis

The data were entered, cleaned, and analyzed using the Statistical Package for Social Sciences (SPSS) version 25. The findings are presented in texts and tables.

### Data quality control

The questionnaire was pretested with 5% of the sample size at Alert Hospital, and amendments were made to the tool. The data were checked for completeness and consistency, and training was provided for the data collectors.

### Ethical considerations

Ethical clearance was obtained from the Atlas College of Health Sciences Ethics Committee. The study also received approval from Zewditu Memorial Hospital. Written informed consent was obtained from all participants. The information was kept confidential. All methods were performed in accordance with the relevant guidelines and regulations, including the Declaration of Helsinki.

### Operational definitions

#### Good knowledge

Respondents who answered 75% or more of the knowledge-related questions correctly (18).

#### Favorable attitude

Respondents who answered more than 75% of the attitude-related questions positively (18).

#### Good practices

Respondents who answered more than 75% of the practice-related questions correctly (19).

## Results

### Sociodemographic characteristics

Most participants (118, 55%) were male and between 20 and 50 years old (46.2%). Approximately thirty percent of the participants had completed primary school, and 68.2% were city dwellers. In addition, 64% of the respondents were married (Table 1).

**Table 1:** Sociodemographic characteristics of the respondents at Zewditu memorial hospital, 2024.

Variables	Number *n*	Percent
Sex		
Male	118	55
Female	96	45
Age in years		
<20	8	2.5
20–50	147	46.2
50–65	44	13.8
>65	15	4.7
Residence		
Urban	146	68.2
Rural	68	31.8
Marital status		
Single	23	10.7
Married	137	64
Divorced	27	12.6
Widowed	27	12.6
Level of education		
No-formal education	26	12.2
Primary	63	29.4
Secondary	63	29.4
College and above	62	29.0

### Knowledge about halitosis among dental patients

**Figure 1:**
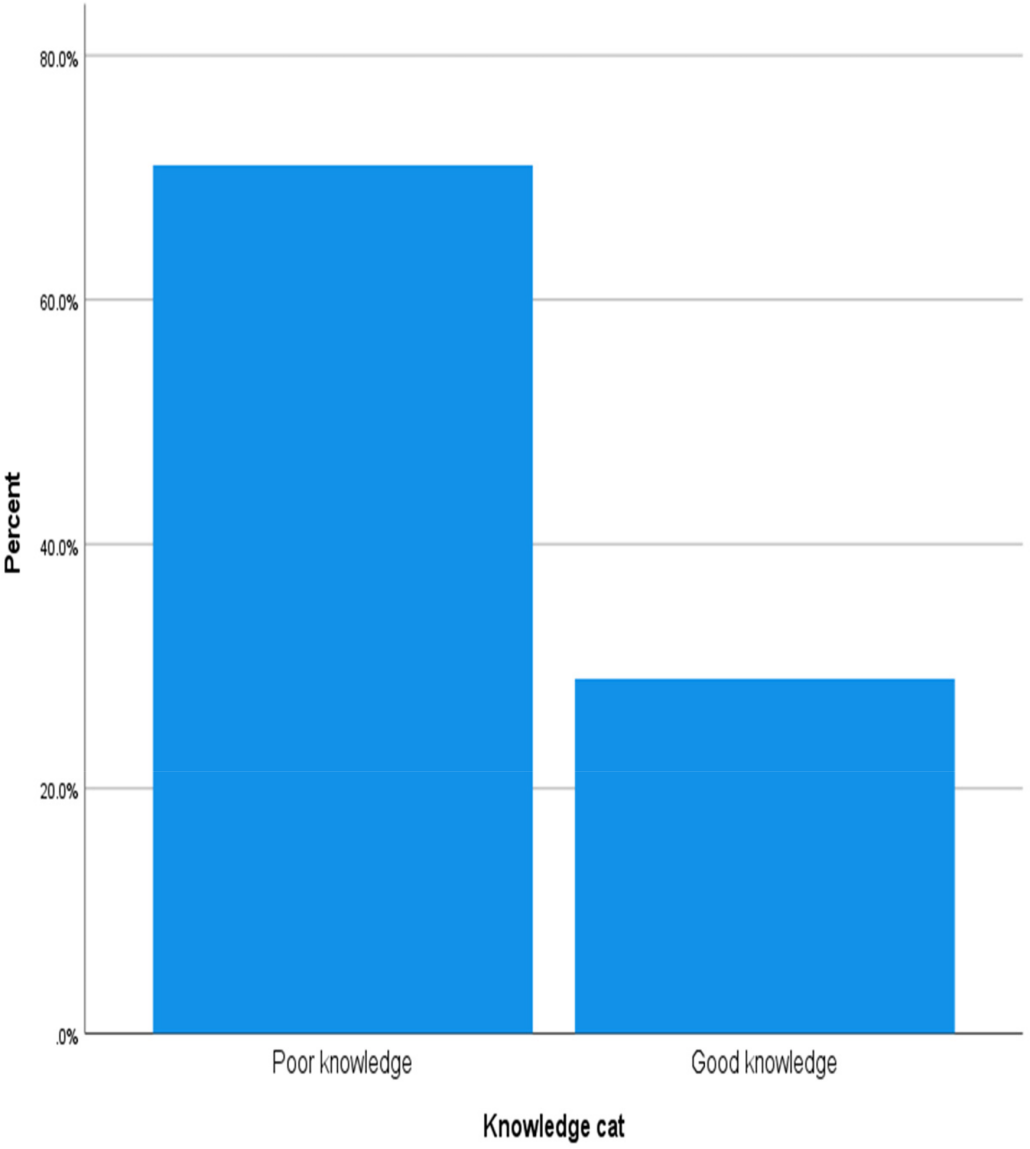
Knowledge status of dental patients at ZMH, 2024.

Among the total number of participants, 91 (42.5%) responded “I don't know” that tooth decay can cause halitosis, whereas 94 (43.9%) responded that plaque can. In addition, only 35.5% of the participants reported that food debris can cause halitosis, and 40% reported that gastrointestinal diseases and tonsillitis can cause halitosis (Table 2). Overall, 71% of the participants had poor knowledge about halitosis (Figure 1).

**Table 2:** Knowledge about halitosis among dental patients at ZMH, 2024.

Variables	Number *n*	Percent
Do you think dental decay can cause halitosis?		
No	38	17.8
I don't know	91	42.5
Yes	85	39.7
Do you think dental plaque can cause halitosis?		
No	36	16.8
I don't know	84	39.3
Yes	94	43.9
Do you think tongue coating can cause halitosis?		
No	35	16.4
I don't know	80	37.4
Yes	99	46.3
Dry mouth can cause halitosis.		
No	38	17.8
I don't know	92	43
Yes	84	39.3
Do you think food debris can cause halitosis?		
No	45	21
I don't know	93	43.5
Yes	76	35.5
Do you think diseases gastrointestinal diseases, tonsillitis can cause halitosis		
No	34	15.9
I don't know	95	44.4
Yes	85	39.7

### Attitude toward halitosis

Overall, the participants had an unfavorable attitude toward halitosis (68.7%). Among the participants, 85 (39.7%) agreed that eating garlic can cause halitosis, whereas 89 (41.6%) agreed that people who have halitosis suffer from social interaction (Table 3).

**Table 3:** Attitudes toward halitosis among dental patients at ZMH, 2024.

Variables	Number *n*	Percent
Brushing teeth irregularly can cause halitosis.		
Disagree	34	15.9
Neutral	87	40.7
Agree	93	43.5
Eating garlic can cause halitosis.		
Disagree	43	20.1
Neutral	86	40.2
Agree	85	39.7
People who have halitosis suffer from social interaction.		
Disagree	34	15.9
Neutral	91	42.5
Agree	89	41.6
Two partners put down their relationship due to halitosis.		
Disagree	33	15.4
Neutral	90	42.1
Agree	91	42.5
Cigarette smoking can cause halitosis.		
Disagree	56	26.2
Neutral	80	37.4
Agree	78	36.4
Good oral hygiene can remove halitosis.		
Disagree	49	22.9
Neutral	87	40.7
Agree	78	36.4
Attitude status		
Unfavorable attitude	147	68.7
Favorable attitude	67	31.3

### Practices toward halitosis

Among the participants, 87 (40.7%) reported using oral hygiene practices to prevent bad breath. Eighty-nine (41.6%) participants reported brushing their tongue, and 43% used toothpaste regularly to prevent bad breath. However, only 38.8% of the participants visited a dentist at least once a year for professional cleaning. Overall, 57 (26.6%) of the participants had good halitosis practices (Table 4).

**Table 4:** Practices toward halitosis among dental patients at ZMH, 2024.

Variables	Number *n*	Percent
Do you practice oral hygiene to avoid halitosis?		
No	127	59.3
Yes	87	40.7
When brushing your teeth, do you also brush your tongue?		
No	34	15.9
Sometimes	91	42.5
Yes	89	41.6
Do you always rinse your mouth with water after eating?		
No	32	15.0
Sometimes	91	42.5
Yes	91	42.5
Do you use toothpaste to avoid bad breath?		
No	35	16.4
Sometimes	87	40.7
Yes	92	43
Do you also brush your lingual and palatal surface while brushing		
No	33	15.4
Sometimes	91	42.5
Yes	90	42.1
How often do you visit a dentist?		
Only when I am sick	30	14
Once in a year	83	38.8
Twice or more in a year	101	47.2
Practice status		
Poor practice	157	73.4
Good practice	57	26.6

## Discussion

The results of this study provide valuable insights into the knowledge, attitudes, and practices regarding halitosis among dental patients at Zewuditu Memorial Hospital.

The findings of this study revealed a lack of understanding among participants regarding the common causes of halitosis, such as poor oral hygiene (39.7%), certain foods (35.5%), and medical conditions (39.7%), with only 29% demonstrating good knowledge of halitosis. This aligns with a study conducted in Jordan (20), which reported a low awareness rate of 20.5%, and Saudi Arabia (9), which reported limited knowledge regarding halitosis. In contrast, a study in Karachi, Pakistan (10), indicated that 54.7% of participants had good knowledge of halitosis, and a survey conducted in Saudi Arabia (21) revealed a high level of knowledge about halitosis, highlighting a significant disparity. This study revealed that 46.3% of the participants knew that tongue coating and 35.5% knew that food debris could cause halitosis, which is consistent with a study conducted in India (12). Differences in awareness of halitosis may be influenced by contextual factors such as cultural attitudes toward oral health, the effectiveness of educational initiatives, and access to information in different regions. Methodological differences, including differences in study design, sample size, demographic characteristics, and survey instruments, may also affect knowledge levels and contribute to discrepancies in results. The low awareness of halitosis highlights the need for targeted public health initiatives and education programs to improve understanding, particularly in areas with limited knowledge.

This study revealed that 31.3% of the respondents had favorable attitudes toward halitosis, which is lower than the 52.5% reported in a study conducted in India (12). This discrepancy may stem from variations in the methods used to assess attitudes and differences in the populations surveyed. Additionally, in a survey performed among patients attending dental OPD in a private dental hospital in Karachi, patient attitudes toward halitosis were not satisfactory (10). Furthermore, 41.6% of the participants acknowledged that halitosis can adversely affect an individual's social life. This finding highlights the social impact of halitosis and suggests that individuals are aware of its potential impact on personal relationships and social interactions. The limited positive attitudes toward halitosis indicate a gap in public understanding that could lead to negative social consequences for those affected. This highlights the importance of targeted public health initiatives to improve awareness and attitudes toward halitosis. Educational programs that address the stigma associated with halitosis and provide information on the causes, prevention, and treatment of halitosis could be beneficial.

The findings of this study indicate that while a significant portion of the participants engaged in practices aimed at combating halitosis, 40.7% reported receiving regular oral care, 42.5% used mouthwash, and 38.8% visited the dentist annually for professional cleaning, while only 26.6% had good practice toward halitosis. This suggests that many individuals may not employ effective strategies to combat bad breath despite engaging in oral hygiene practices. These results align with studies conducted in Nepal (14) and Benin (16), which reported unsatisfactory practices toward halitosis. This consistency between studies suggests a broader problem that may be prevalent in different populations. Several factors may contribute to low practices of halitosis, such as a lack of knowledge about the specific causes of halitosis to address the underlying issues. While regular oral care and the use of mouthwash are important, they may not be enough if they do not address the root causes of bad breath, such as poor dental hygiene, certain foods, or underlying medical conditions. The low percentage of participants' practices suggests that more education on oral health practices is needed. Public health initiatives should promote general oral hygiene and provide specific information about halitosis, its causes, and effective treatment strategies.

This study is not without flaws and has limitations. One major concern is the potential for social desirability bias, which could affect the accuracy of participants' responses. In addition, conducting the study in a single hospital further limits the generalizability of the results. Moreover, the study did not examine the reasons for participants' inadequate knowledge, attitudes, and practices regarding halitosis. To address these gaps, future research should prioritize qualitative studies that explore participants' knowledge, attitudes, and practices (KAPs) regarding halitosis to provide deeper insights into the observed discrepancies.

## Conclusions

In general, knowledge, attitudes, and practices regarding halitosis among adult dental patients at Zewditu Memorial Hospital are inadequate. Addressing halitosis in Ethiopia requires a multifaceted approach focusing on education about oral hygiene, improving access to dental care, and understanding the causal factors contributing to halitosis.

## Data Availability

The original contributions presented in the study are included in the article/Supplementary Material, further inquiries can be directed to the corresponding author.

## Footnotes

^1^Anjulo AL, Siyoum MB. *Assessment of knowledge, attitude, and practice toward causes & prevention of bad breath in Jimma University Students of Faculty of Business and Economics Campus, Jimma, Ethiopia, 2015* (unpublished student thesis). Jimma University repository (2015).
